# Risk for Disability and Poverty Among Central Asians in the United States

**DOI:** 10.5195/cajgh.2015.220

**Published:** 2016-02-18

**Authors:** Carlos Siordia, Athena K. Ramos

**Affiliations:** 1 Center for Aging and Population Studies, Graduate School of Public Health, University of Pittsburgh, PA;; 2 Center for Reducing Health Disparities, College of Public Health, University of Nebraska, NE

**Keywords:** disability, poverty, American Community Survey, Public Use Microdata Sample, former USSR, Central Asia

## Abstract

Understanding the disability-poverty relationship among minority groups within the United States (US) populations may help inform interventions aimed at reducing health disparities. Limited information exists on risk factors for disability and poverty among “Central Asians” (immigrants born in Kazakhstan, Uzbekistan, and other Central Asian regions of the former Soviet Union) in the US. The current cross-sectional analysis used information on 6,820 Central Asians to identify risk factors for disability and poverty. Data from the 2009–2013 Public Use Microdata Sample (PUMS) file from the American Community Survey (ACS) indicate that being married, non-Latino-white, and having higher levels of educational attainment are protective against disability and poverty. In contrast, older age, residing in the Middle Atlantic geographic division, and having limited English language ability are risk factors for both disability and poverty. Research should continue to develop risk profiles for understudied immigrant populations. Expanding knowledge on the well-being of Central Asians in the US may help impact public health interventions and inform health policies.

In the United States (US) and across the world, having the ability to overcome psycho-social and physical barriers for independent living can influence quality of life.^1^ The differently abled (i.e., disabled) and economically deprived (i.e., poor) are at greater risk for adverse health.^2^ Although low-income minorities should not be defined as being “trapped in an inescapable cycle of poverty,”^3^ it should be noted that both the physically and economically disadvantaged individuals do face unique challenges for resisting exposures that may lead to disability.^4^ Unfortunately, the relationship between disability and poverty (i.e., the disability-poverty nexus) remains under-researched among smaller immigrant groups.^5^–^7^ This investigation fills a gap in the disability-poverty nexus literature by focusing on the Central Asian population of the US.

Although research on the disability-poverty nexus on minorities has been conducted,^8^–^11^ it is difficult to find peer-reviewed publications in scientific journals that focus on the group that may be called the Central Asian population of the US. Although explained more technically below, “Central Asians” in this investigation were selected based on place of birth. The US Census Bureau only defines “South Central Asians.”^12^ This is the first publication to define Central Asians as immigrants who report being born in Kazakhstan, Uzbekistan, former USSR, or other South Central Asia, not specified. The specific aim of this investigation was to identify risk factors for disability and poverty in the Central Asian immigrant population in the US.

## Methods

### Data

This cross-sectional analysis used data from the American Community Survey (ACS), 5-year (2009–2013) Public Use Microdata Sample (PUMS) file. Data from the ACS is important for public health research because it may be used to influence policy aimed at providing services for disabled individuals. For example, ACS data influenced the distribution of US federal dollars to local governments in 2008 by affecting the distribution of $562.2 billion in grant funds.^13^ The use of publicly available ACS data does not require institutional review board approval. The same data source has been used before to delineate the prevalence of disability and poverty in other minority and immigrant populations in the US.^14^–^16^ ACS PUMS data may be one of the few data sources that has the capacity to provide information on the demographic, geographic, and health profiles of the Central Asian population in the US.

### Central Asians

The sample of 6,820 Central Asians was selected using Place of Birth (POB). The US Census Bureau only provides an official definition of South Central Asia which includes places like India and Iran.^12^ The analysis labeled US immigrants born in Kazakhstan, former USSR, Uzbekistan, and other South Central Asia, not specified as “Central Asians.” Although this data source does not allow researchers to identify immigrants who report being born in places like Kyrgyzstan, Tajikistan, and Turkmenistan, immigrants who were coded under the “other South Central Asia, not specified” category where included as Central Asians in the analysis. After extensive research, this appears to be the first analysis defining Central Asians as individuals born in Kazakhstan, former USSR, Uzbekistan, and other South Central Asia, not specified while using ACS data. While this grouping scheme is imperfect, limitations of the data source do not allow analysis by specific country of origin.^17^ As with people born in Kazakhstan, Uzbekistan, Kyrgyzstan, Tajikistan, and Turkmenistan, identifying immigrants in the US from recently transformed regions of the world is difficult.^18^ For simplicity, the selected sample is referred to as Central Asians. In addition to POB, individuals were only included in the sample if they resided within the contiguous US.

### Disability

Central Asians were labeled as “disabled” if they reported a ‘yes’ to one or more of the following questions: Is this person deaf or does he/she have serious difficulty hearing?; Is this person blind or does he/she have serious difficulty seeing even when wearing glasses?; Because of a physical, mental, or emotional condition, does this person have serious difficulty concentrating, remembering, or making decisions?; Does this person have serious difficulty walking or climbing stairs?; Does this person have difficulty dressing or bathing?; Because of a physical, mental, or emotional condition, does this person have difficulty doing errands alone such as visiting a doctor’s office or shopping? Limitations and benefits of using these questions have been discussed in detail previously;^2^,^8^ however, responses to these questions may be utilized to identify perceived ability to perform physical functional tasks in daily living.^9^ It is important to note subjectively assessed physical function faces measurement bias because study participants are asked to self-evaluate their health and rely on their memory for reporting the data. In addition, in the ACS, the majority of individuals are assigned their disability status through a proxy-report.^16^ Despite these limitations, the ACS is the most reliable and largest data source for delineating the disability of hard-to-reach populations in the US.

### Poverty

The ACS PUMS file contains the “income-to-poverty ratio” (IPR) measure. The IPR shows at what ‘level of poverty’ an individual is—e.g., being at or below a 100 IPR indicates that this person is in-poverty status. For example, in fiscal year 2013, the poverty threshold for a family of four was $23,550. Thus, all members of a family with a total household income of $23,550 would be assigned an IPR of 100 and be coded as being “in-poverty.” If the family reports a total household income of $11,775, each family member would be assigned an IPR of 50 and be coded as being in “deep-poverty.” If a family reports a total household income of $35,325, then each family member would be assigned an IPR of 150 and be coded as being “near-poverty.” In this analysis, a person is classified to be in-poverty if they have an IPR of 150 or below—an approach used to account for the fact that federal poverty thresholds do not account for geographical heterogeneity in cost-of-living.

### Statistical approach

All data management and analysis was conducted using SAS® 9.3 software. Although population-weights are provided in the data, they were not used because generalizing information from 6,820 individuals to about 149,473 Central Asian immigrants would result in population estimates with very large standard errors. That is, the 95% confidence limits around the population estimates would be large enough to render population-weighted estimates uninformative. Descriptive statistics for the analytic sample are presented. Non-population-weighted multivariable logistic regressions were used to model the likelihood of being disabled or in-poverty while adjusting for age, sex, marital status, race, ethnicity, naturalization status, ability to speak English, educational attainment, and geographic division of residence. Variables were coded using common approaches in socio-epidemiological research on poverty and disability.

## Results

### Participant characteristics

Our sample was 54% female, 59% married, 83% non-Latino-white, 60% US citizens (95.6% by naturalization, 4.4% by being born to parents with US citizenship), 18% lacked health insurance coverage, and 24% were unable to speak English ‘well’ or ‘at all’ (Table 1). Participants in this sample were aged 40.6±19.9, 36% reported high school education or below, and the majority (36%) of participants resided in the Middle Atlantic geographic division of the US. About 13% of the sample reported a disability and about 30% may be classified as being in-poverty (i.e., IPR < 150). The low level of disability in the sample may be partially explained by the plausibility that the selection process influences who emigrates from Central Asia

### Risk factors for disability

Several factors were identified as risk factors for disability. For example, naturalized citizens were twice as likely to be disabled compared to those who report not being a citizen or naturalized (Table 2). Those who spoke English not well or not at all were over three times as likely to be disabled as those who spoke English very well or well. Each increase in age (by year) was associated with a 7% increase in the likelihood of being disabled. In addition, residing in the Middle Atlantic geographic division was associated with a 20% greater likelihood of being disabled than residing in other divisions. Education and marital status were associated with lower odds of being disabled. For example, married Central Asians were 64% less likely to be disabled than non-married people. Each increase in educational attainment category was associated with a 7% decrease in the likelihood of being disabled.

### Risk factors for poverty

The regression (Table 3) indicates that being married, non-Latino-white, and a naturalized citizen was associated with a 52%, 33%, and 56%, respectively, lower odds of being in-poverty. Each additional increase in educational attainment category was associated with a 4% reduction in the likelihood of being in-poverty. In contrast, residing in the Middle Atlantic division reduced the odds of poverty by 16% while speaking English not well or not at all significantly increased the odds of poverty almost three-fold.

## Discussion

High levels of educational attainment and being married were found to be consistently protective against disability and poverty. In contrast, older ages, residing in the Middle Atlantic geographic division, and speaking English not well or not at all were consistently found to be risk factors for disability and poverty. More complex is the fact that being a naturalized citizen is simultaneously a risk factor for disability and protective against being in-poverty. It may be said that a “Central Asian Immigrant Paradox” is found, where those at greater risk for economic disadvantage are also at lower risk for disability. This is paradoxical because for most non-immigrant groups, greater risk for poverty is frequently accompanied by greater risk for disability.^19^,^20^ Future research should seek to disentangle plausible explanations for the Central Asian Immigrant Paradox.

This study has some limitations including the inability to obtain and/or identify data from individuals from some of the countries within the region such as Kyrgyzstan, Tajikistan, and Turkmenistan as previously described. There is an inherent selection bias in participation in government administered surveys which may partially be affected by legal status. Furthermore, subjectively assessing disability through recall and self-report may affect the validity of the results. It is possible that individuals born in Central Asia and who have the ability and the means to immigrate to the US come from family units with social and economic resources. The low prevalence of disability in the analytic sample may be partially explained by the fact that about two-thirds of the sample is below age 47 or due to ‘healthy immigrant selectivity.’^9^

Objective measurements for disability are needed to supplement these results. Poverty is assessed using only economic measures. Additional measures for both poverty and health status are also needed to be able to assess the impact of disability on poverty and health status.

The cross-sectional analysis provides empirical evidence on the understudied Central Asian population in the US. Findings from this analysis should be generalized with caution to the community-dwelling immigrants born in Kazakhstan, former USSR, Uzbekistan, other South Central Asia, not specified and who resided within the US mainland during the survey period. A multitude of challenges remain for intervening on the relationship between poverty and disability.^21^ Future research should be sensitive to poverty indicators focusing on practical matters such as quantifying the risk for poverty among individuals related to someone with a disability.^22^ For immigrants residing in the US, like Central Asians, disability and poverty have the potential to become entangled with other forms of oppressions.^23^ Research should continue to explore if and how immigrants from Central Asia residing in the US retain their language, identity, health, family structures, and economic well-being.

## Figures and Tables

**Table 1.** Demographics of 6,820 Central Asians living within the US

**Characteristic**	**N (%)**
Disabled	884 (13)
Female	3,667 (54)
Married	3,990 (59)
Non-Latino-White	5,630 (83)
US citizen	4,089 (60)
Has no health insurance coverage	1,204 (18)
Speaks English not well/at all^5^	1,658 (24)
Age group	
Age ≤ 17	839 (12)
Age 18–27	1,171 (17)
Age 28–37	1,216 (18)
Age 38–47	1,123 (16)
Age 48–57	957 (14)
Age ≥ 58	1,514 (22)
Education	
≤ 8^th^ grade	1,026 (15)
9^th^ – 12^th^ grade*	499 (7)
High school	968 (14)
Some College	1,319 (19)
≥ Bachelor’s degree	3,008 (44)
Poverty level	
Deep poverty (≤50)	542 (8)
In-poverty (51–100)	798 (12)
Near poverty (101–150)	697 (10)
Above near-poverty (151–200)	581 (9)
Out of poverty (≥200)	4,202 (62)
Location	
New England	448 (7)
Middle Atlantic	2,453 (36)
East North Central	571 (8)
West North Central	203 (3)
South Atlantic	950 (14)
East South Central	97 (1)
West South Central	295 (4)
Mountain	431 (6)
Pacific	1,372 (20)

* *Note*. Participants in the 9^th^–12^th^ grade education group did not complete high school.

**Table 2.** Multivariable logistic regression predicting the likelihood of being disabled

	OR	95% CI	
Female	0.99	0.83, 1.18	
Married	0.37	0.30, 0.44	***
Non-Latino-White	0.84	0.62, 1.13	
Citizen or naturalized	2.01	1.60, 2.52	***
Speaks English not well/at all	3.25	2.67, 3.97	***
Age	1.07	1.07, 1.08	***
Education	0.93	0.92, 0.95	***
Resides in Middle Atlantic division	1.20	1.00, 1.43	*

* p<0.05,

** p<0.01,

*** p<0.001

**Table 3.** Multivariable logistic regression predicting likelihood of being in poverty

	OR	95% CI	
Female	1.00	0.89, 1.12	
Married	0.48	0.42, 0.54	***
Non-Latino-White	0.67	0.57, 0.78	***
Citizen or naturalized	0.44	0.39, 0.50	***
Speaks English not well/at all	2.86	2.47, 3.30	***
Age	1.01	1.01, 1.02	***
Education	0.96	0.95, 0.97	***
Resides in Middle Atlantic division	1.16	1.03, 1.31	**

* p<0.05,

** p<0.01,

*** p<0.001
